# QTL and Candidate Gene Identification for Silique Length Based on High-Dense Genetic Map in *Brassica napus* L.

**DOI:** 10.3389/fpls.2019.01579

**Published:** 2019-11-29

**Authors:** Hui Wang, Qamar U. Zaman, Wenhui Huang, Desheng Mei, Jia Liu, Wenxiang Wang, Bingli Ding, Mengyu Hao, Li Fu, Hongtao Cheng, Qiong Hu

**Affiliations:** ^1 ^Oil Crops Research Institute of the Chinese Academy of Agricultural Sciences/Key Laboratory for Biological Sciences and Genetic Improvement of Oil Crops, Ministry of Agriculture and Rural Affairs, Wuhan, China; ^2 ^Graduate School of the Chinese Academy of Agricultural Sciences, Beijing, China

**Keywords:** oilseed rape, silique length, quantitative trait loci, high-dense genetic map, double haploid population, re-sequencing

## Abstract

Silique length (SL) is an important yield trait and positively correlates with seeds per silique and seed weight. In the present study, two double haploid (DH) populations, established from crosses Zhongshuang11 × R11 (ZR) and R1 × R2 (RR), containing 280 and 95 DH lines, respectively, were used to map quantitative trait loci (QTL) for SL. A high-dense genetic map from ZR population was constructed comprising 14,658 bins on 19 linkage groups, with map length of 2,198.85 cM and an average marker distance of 0.15 cM. Genetic linkage map from RR population was constructed by using 2,046 mapped markers anchored to 19 chromosomes with 2,217-cM map length and an average marker distance of 1.08 cM. Major QTL *qSL_ZR_A09* and *qSL_RR_A09b* on A09 were identified from ZR and RR populations, respectively. Both QTL could be stably detected in four environments. QTL *qSL_RR_A09b* and *qSL_ZR_A09* were located on 68.5–70.8 cM and 91.33–91.94 cM interval with R^2^ values of 14.99–39.07% and 15.00–20.36% in RR and ZR populations, respectively. Based on the physical positions of single nucleotide polymorphism (SNP) markers flanking *qSL_ZR_A09* and gene annotation in *Arabidopsis*, 26 genes were identified with SNP/Indel variation between parents and two genes (*BnaA09g41180D* and *BnaA09g41380D*) were selected as the candidate genes. Expression analysis further revealed *BnaA09g41180D*, encoding homologs of *Arabidopsis* fasciclin-like arabinogalactan proteins (FLA3), as the most promising candidate gene for *qSL_ZR_A09*. The QTL identification and candidate gene analysis will provide new insight into the genomic regions controlling SL in *Brassica* napus as well as candidate genes underlying the QTL.

## Introduction

The genetic control of domesticated traits has been studied in various crop plants (Nasyrov, 2003; Aitken et al., 2008), including rapeseed, by quantitative trait loci (QTL) mapping using different pairs of crosses between distinct varieties, for specific traits especially to enhance yield (Udall et al., 2006; Bouchet et al., 2014), oil content (Jin et al., 2007; Rout et al., 2018), and disease resistance (Wu et al., 2019). *Brassica napus* L. (AACC, 2n = 38), as one of the essential oil crops worldwide, is a polyploid crop derived by spontaneous hybridization between *Brassica rapa* (AA, 2n = 20) and *Brassica oleracea* (CC, 2n = 18) (Nagaharu, 1935) <7,500 years ago followed by the process of duplication (Boulos et al., 2014; Bayer et al., 2017). Comparative genomic studies showed that the A genome of *B*. rapa and *B. napus* exhibited collinearity with fewer genomic changes and translocations (Wang et al., 2011). Silique length (SL) is one of the most effective components for yield selection in rapeseed, by which not only seed yield can be increased but also the total oil yield (Samizadeh et al., 2007; Bennet et al., 2011). Information regarding inheritance pattern and gene action for SL is useful in rapeseed breeding program. However, the understanding of silique development in *B. napus* is limited and only few functional genes for SL have been explored due to high ploidy level.

In general, increase of SL may enhance the source of matter, which results in larger seeds (Chay and Thurling, 1989). To increase the yield of *B. napus*, identification of QTL and genes for SL is of importance for the development of cultivars with long siliques and incorporating the trait to improve yield (Youssefy et al., 2012). Number of siliques per plant varied independently on SL (Chay and Thurling, 1989). However, SL is positively correlated with seed weight and seed number per silique (Chen et al., 2007) and could serve as one trait for indirect selection of seeds per silique in the breeding process. As one of the important yield-related traits, SL has been extensively studied in terms of QTL analysis in rapeseed (Shi et al., 2019; Zhang et al., 2011; Li et al., 2014; Fu et al., 2015; Wang et al., 2016; Yang et al., 2017).

For QTL identification, second-generation molecular markers including simple sequence repeats (SSR) and amplified fragment length polymorphism (AFLP) are of low density across the genome, resulting in low-quality mapping (Yang et al., 2017). Advanced next-generation sequencing (resequencing) technologies provide new insight to rapidly identify QTL of interest. High-density genetic maps based on high-throughput SNP markers have the advantages of high throughput, low cost, high resolution, and high positioning accuracy, which can significantly improve the efficiency and precision of genotyping and genetic map construction. QTL for complex agronomic traits such as plant height, flowering time, oil content, and seed weight have been mapped based on high-throughput SNP markers (Yang et al., 2012b; Li et al., 2014; Li et al., 2015; Wang et al., 2015).

Many QTL for SL or seed size have been identified in rapeseed (Zhang et al., 2011; Li et al., 2014; Fu et al., 2015; Liu et al., 2015; Wang et al., 2016; Yang et al., 2017). Eight QTL for SL distributed on A01, A06, A07, A09, and C06 were detected from immortalized F2 (IF2) and double-haploid (DH) populations in different environments (Wang et al., 2018). One major QTL was identified for SL and thousand seed weight on A09 that shared a common linked marker suggesting tightly linked or pleiotropic QTL (Qi et al., 2014). Though many QTL have been detected, only two genes affecting SL and seed size have been cloned and characterized. An auxin response factor gene (*ARF18*) involved in SL and seed weight has been cloned by fine mapping and association analysis (Liu et al., 2015). One 55-aa deletion prevents ARF18 forming homodimer and inhibited the activity of downstream auxin genes (Liu et al., 2015). Another research group identified one major QTL on A09 and then fine-mapped *BnaA9.CYP78A9* as underlying gene for *qSLWA9* locus (Yang et al., 2012b; Shi et al., 2019). A 3.7-kb insertion of a CACTA-like transposable element (TE) in the regulatory region functioned as an enhancer to increase the expression of *BnaA9.CYP78A9* in varieties with long siliques and large seeds (Shi et al., 2019), whereas the insertion was replaced by a 12.3-kb deletion in varieties with short siliques. Interestingly, the physical distance of these two genes is less than 20 kb, suggesting the complex of regulating genes for SL in the A09 chromosome region.

In the present study, we identified QTL for SL by using two independently generated double haploid populations derived from crosses Zhongshuang11 × R11 and R1 × R2. Major QTL on A09 chromosome were stably detected in all environments from both populations, and the contribution rate is between 15.00% and 20.36%. Markers with physical position revealed that the *qSL_ZR_A09* of ZR population may be one novel QTL on A09. Gene structure variation and expression analysis showed that *BnaA09g41180D*, which is homologous to *Arabidopsis FLA3*, may be the best promising genes for SL control. This QTL at the specific locus *qSL.A09* and the information of candidate gene will be valuable for marker-assisted selection (MAS) breeding for higher yield and further identification of functional gene related to yield improvement in rapeseed.

## Materials and Methods

### Plant Materials

Four semi-winter-type inbred lines developed by OCRI-CAAS including Zhongshuang11 (ZS11), R11, R1, and R2 were selected for dissecting the genetic bases of SL in *B. napus*. ZS11 and R1 have long siliques while R11 and R2 have regular SL. Two DH populations were developed from the crosses between ZS11 × R11 and R1 × R2. The DH populations derived from the crosses of ZS11 × R11 and R1 × R2 parents were named as “ZR population” and “RR population”, respectively. In total, 280 and 95 DH lines were generated from ZR and RR populations, respectively.

### Experimental Design and Trait Measurement

The DH populations including DH lines and parents were grown in field using a randomized complete block design with three replications during September to May in Wuhan (114.33°E, 30.50°N), Huanggang (114.87°E, 30.44°N), and Zunyi (106.55°E, 27.42°N) each year (**Supplementary Figure 1)**. Location–year combination was coded as environments. The four environments for ZR population were WH15 (Wuhan, 2014–15), WH16 (Wuhan, 2015–16), WH17 (2016–17), and HG17 (Huanggang, 2016–17). And the four environments for RR DH population were WH13 (Wuhan, 2012–13), WH14 (Wuhan, 2013–14), ZY13 (Zunyi, 2012–13), and ZY14 (Zunyi, 2013–14). Each plot contained three rows and 20 plants in each row. The distance between rows was 33 cm and between plants was 10 cm. At maturation stage when most siliques turned yellow, 10 plants were randomly sampled from the middle row of each plot and considered as representative for trait measurement. Thirty well-developed siliques of the main inflorescence from each plant were measured for SL.

### Statistical Analysis

Analysis of variance (ANOVA) was performed using the procedure MEANS of SAS 9.1 (SAS, 2004) for descriptive phenotypic statistics. The phenotypic *t* test of parents was calculated using the procedure TTEST of SAS 9.1. The broad-sense heritability was calculated as $h^{2}=\sigma_{g}^{2}/(\sigma_{g}^{2}+\sigma_{ge}^{2}/n+\sigma_{e}^{2}/nr)$ , where $\sigma_{g}^{2}$ is genetic variance, $\sigma_{ge}^{2}$ is the interaction variance of genotype with environment, $\sigma_{e}^{2}$ is error variance, *n* is the number of environments, and r is the number of replications. Components of variance ( $\sigma_{g}^{2}$ , $\sigma_{ge}^{2}$, and $\sigma_{e}^{2}$ ) were estimated using the GLM procedure of SAS 9.1.

### SNP Genotyping and Linkage Map Construction of RR Population

The *Brassica* 60 K SNP BeadChip Array was used to genotype 95 RR DH lines and two parental lines. Genetic linkage map of RR population has been constructed in our previous study (Liu et al., 2016).

### SNP Genotyping and Linkage Map Construction of ZR Population

#### Sequencing Library Construction and High-Throughput Sequencing

The genomic DNA of 280 ZR DH lines and parental lines (ZS11 and R11) was extracted from the leaves by CTAB method. The genomic DNA was sheared into ∼500-bp fragments using the S2/E210 Ultrasonicator (Covaris, USA). About 2 µg genomic DNA from each line was prepared for sequencing library construction. Sequencing library was constructed by "NEB DNA Library Prep Kit" according to the manufacturer’s instructions. The sequencing libraries were constructed by terminal repair, then added 3′A and a sequencing linker. Sequence data were generated by Illumina HiSeq 4000 (San Diego, California, USA) with paired-end (PE150). Sequence quality and adaptor trimming was conducted by SOAPnuke 1.4 (BGI, Shenzhen, China).

#### SNP Identification and Genotyping

The raw reads which came from the original data of the Illumina HiSeq™ sequencing platform were processed using a series of in-house C-scripts for quality control (QC). During QC procedures, low-quality reads were filtered out and clean reads were obtained. Burrows–Wheeler Aligner (BWA) (Li and Durbin, 2009) software was used to align the clean reads of each sample against the *B. napus* genome (https://www.ncbi.nlm.nih.gov/assembly/GCF_000686985.1/). SAMtools (McKenna et al., 2010) software was used to convert the alignment files to bam files and remove duplicate reads. The bam files were filtered by GATK toolkit and potential SNPs between all lines and the genome were detected. The SNPs identified between the parents were considered as polymorphic for a subsequent bin calling. To reduce false-positive SNPs caused by sequencing errors, the base support for each SNP in the parents and DH lines were required to be 4 or more (and no more than 1,000). All SNP markers between the parents were classified into four segregation patterns (aa × bb, hk × hk, lm × ll, and nn × np). For the DH populations, the segregation patterns aa × bb were selected for construction of genetic map.

#### Bin Map and Linkage Map Construction

The recombinant bin map based on SNP markers was constructed by a modified slide window method which was developed by Huang’s lab (Huang et al., 2009). The genotype of each window was described as a window of 15 SNPs and a step size of 1 SNP. Windows containing more than 13 "aa" or "bb" types were genotyped as aa or bb, respectively. Fifteen adjacent SNP intervals with the same genotype across the entire ZR population were combined into a recombination bin. The linkage map was constructed from the recombination bins serving as genetic markers using the HighMap program (Liu et al., 2014). The Kosambi mapping function was used to calculate the genetic distance between the markers (Kosambi, 2016).

#### QTL Mapping and SNP Bin Map for ZR Population

Data which were collected in different years were considered as an independent environment. WinQTLCart2.5 (Wang, 2006) was used to conduct QTL mapping of ZR and RR populations with composite interval mapping method and Model 6 Zmapqtl procedure. A 1,000 permutation test of shuffling the phenotypic means with genotypic means was performed to calculate the significance threshold of the test statistics for one QTL (Doerge and Rebaï, 1996). The parameters of control markers, windows size, and walk speed were fixed to 5, 10, and 1 cM, respectively. The marker distance was set to 5 cM to determine a significant QTL. The percentage of phenotypic variance explained by QTL was predicted by the highest probability peaks. For SL trait, QTL repeatedly detected at the same location of a chromosome with the identical direction of genetic effects were considered as the same QTL.

### Candidate Genes Analysis

To identify candidate genes for SL, genetic and physical map integration of QTL confidence intervals was performed. We used the 1-LOD confidence intervals as the QTL confidence intervals, and the flanking sequences of the SNP markers in QTL confidence intervals were mapped to the *B. napus* reference genome (http://www.genoscope.cns.fr/brassicanapus) using BLAST tool to project QTL on the physical map. Candidate genes were identified based on the annotation of the *Arabidopsis* genome and physical positions of SNP markers in the reference *B. napus* genome.

### Expression Analysis

Total RNA was extracted with Trizol Reagent (Invitrogen, America). Reverse transcription was performed according to the instruction of FastQuant RT Kit (Tiangen, China). RT-PCR was performed as described previously using the primers listed in **Table S4**. The expression level of *actin* gene in *B. napus* was used to standardize the RNA sample for each RT-PCR. The reaction was conducted using the following program: 5 min at 95°C, 35 cycles of 30 s at 95°C, 40 s at 56°C, and 1 min at 72°C.

## Results

### Phenotypic Variation of SL in Parents and DH Lines

**Figure 1:**
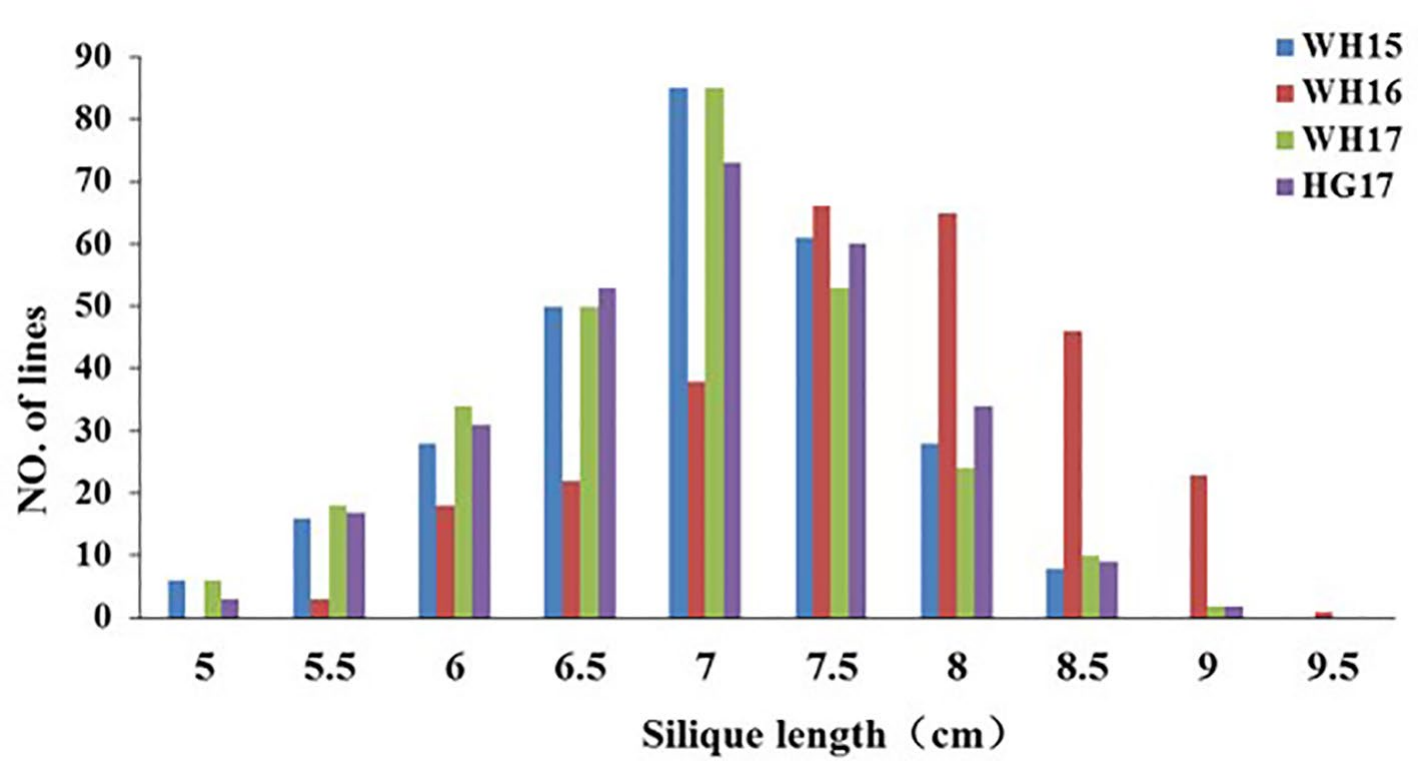
Distribution of silique length in the ZR population derived from the cross Zhongshuang 11 × R11. WH15, WH16, WH17, and HG17 represent four environments with *different colors*. WH15: Wuhan, 2014–15; WH16, Wuhan, 2015–16; WH17, Wuhan, 2016–17; and HG17, Huanggang, 2016–17.

The 280 ZR DH, 95 RR DH lines, and their corresponding parents were grown in winter–spring seasons. The field performance of DH populations for SL across the years was analyzed. The parents bearing long siliques showed maximum SL of 8.33 ± 0.37 cm (ZR crosses) in WH16, and minimum SL of 6.65 ± 0.21 cm (RR crosses) was exhibited in WH14 (**Table 1** and **Figure 1**). One-way ANOVA across environments indicated that genotypes (representing established DH populations and their parents), growing environments, and genotype–environment (G × E) interaction had a highly significant effect on SL (*P* < 0.0001) (**Table 2**). The broad-sense heritability (*h* ^2^) for SL was 94.53% in RR and 97.30% in ZR populations. The normal distribution of SL variation in both DH populations suggested that SL trait is controlled by multiple genes (**Table 1**).

**Table 1:** Descriptive statistics on silique length SL (in millimeters) of parents and double-haploid (DH) lines in RR and ZR populations.

Population (Environment)	Parents		DH lines	
	P1	P2	Mean ± SD	Range
RR (WH13)	7.68 ± 0.05A	6.10 ± 0.16B	7.01 ± 0.98	4.79–9.10
RR (ZY13)	7.94 ± 0.18A	6.70 ± 0.26B	7.18 ± 0.92	4.98–9.56
RR (WH14)	6.65 ± 0.21A	5.71 ± 0.09B	6.23 ± 0.81	4.24–8.16
RR (ZY14)	7.90 ± 0.04A	6.55 ± 0.03B	6.54 ± 0.82	4.91–8.35
ZR (WH15)	7.76 ± 0.43A	6.40 ± 0.13B	6.95 ± 0.73	4.87–8.58
ZR (WH16)	8.33 ± 0.37A	7.50 ± 0.08B	7.63 ± 0.82	5.61–9.50
ZR (WH17)	7.69 ± 0.13A	6.24 ± 0.58B	6.89 ± 0.80	5.05–9.18
ZR (HG17)	7.62 ± 0.14A	6.12 ± 0.10B	6.98 ± 0.77	4.92–8.93

Within the same population (environment), different uppercase letters after numbers indicate a significant difference at the 0.01 probability level among the two parents based on t test, respectively. P1 represents female parents (R1 in RR and ZS11 in ZR populations, respectively). P2 represents male parents (R2 in RR and R11 in ZR populations, respectively).

**Table 2:** ANOVA and broad-sense heritability (*h* ^2^) of silique length (SL) in RR and ZR populations.

Trait	Source	*df*	Sum of square	Mean square	*F* value	*P*	*h* ^2^ (%)
RR	Genotype	94	705.25	7.50	68.80	<0.0001	94.53
	Environment	3	158.84	52.95	485.51	<0.0001	
	Genotype × environment	282	115.16	0.42	3.85	<0.0001	
	Error	721	78.63	0.11			
ZR	Genotype	279	1,850.44	6.63	88.14	<0.0001	97.30
	Environment	3	291.24	97.08	1,290.17	<0.0001	
	Genotype × environment	837	151.99	0.18	2.41	<0.0001	
	Error	2,178	163.88	0.08			

### High-Density SNP Genetic Map Construction

#### Re-Sequencing and Genotyping

The RR population map contained 2,046 marker loci with an average marker density of 1.08 cM along a 2,217.2-cM genetic map. This linkage map was described in more detail elsewhere (Liu et al., 2016). For ZR population, over 609 Gb of clean data (∼2 billion reads) was generated for the parental lines and 280 DH lines. In parental lines of ZS11 and R11, 50.78 Gb clean data (∼170 million reads) and 62.51 Gb clean data (∼209 million reads) were produced, respectively. While 495.75 Gb clean data (∼16.53 billion reads) was generated for the 280 DH lines with more than 93% and 86% of the bases higher than Q20 and Q30, respectively. The average depth coverage per DH line ranged from 1.10× to 2.10×, generated 3.50–8.41 million reads with 150-bp length for each line, whereas in parents the average depth coverage was 36.33× (ZS11) and 44.76× (R11) (**Supplementary Datasheet**). A total of 3,359,647 SNPs were detected between the two parental lines, and 2,342,750 of those remained after removing low-quality SNPs. Of the 2,342,750 high-quality SNPs, 1,122,653 SNPs were aa × bb type and used for genetic mapping.

#### Linkage Map of Recombination Bins

**Figure 2:**
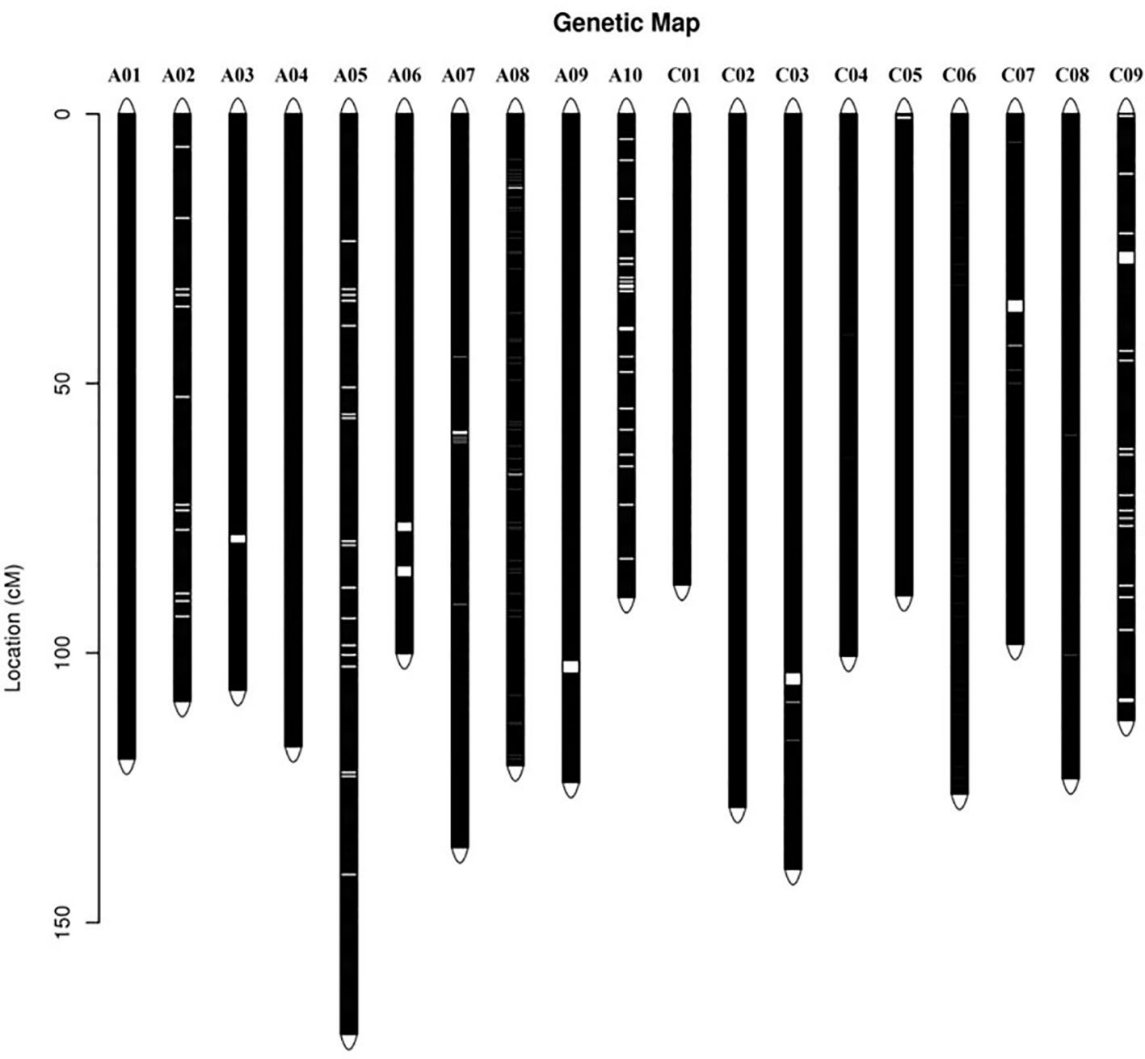
Distribution of single nucleotide polymorphism (SNP) markers on the ultra-dense genetic map. *Bars* on the linkage groups indicate SNP markers, while the *y*-axis determines the genetic distance in centimorgans (cM).

**Figure 3:**
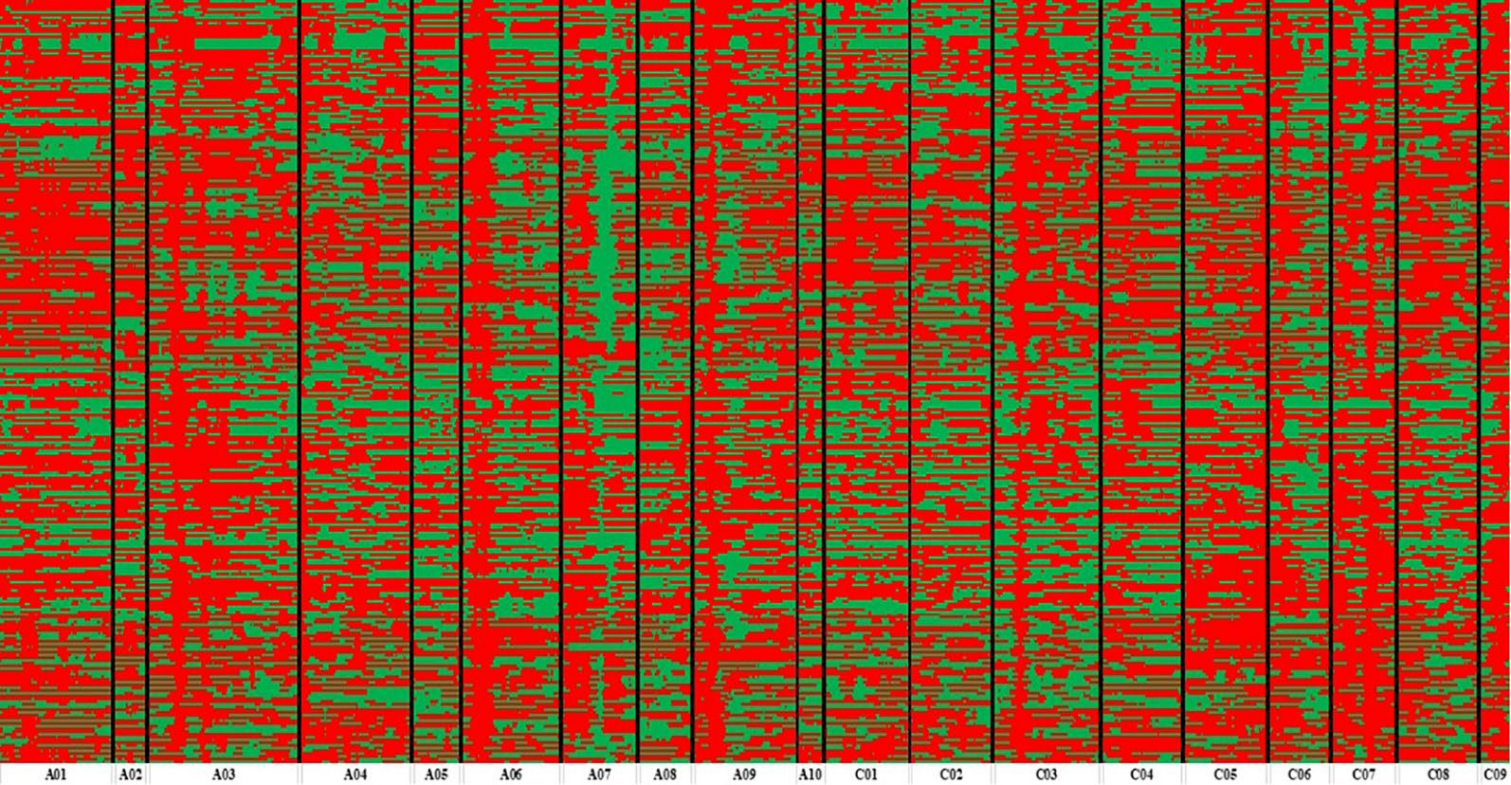
Bin map of ZR population. Bin map consists of 14,658 bins inferred from re-sequencing-based high-quality single nucleotide polymorphisms (SNPs). *Different colors* represent different genotypes: *red color* exhibits ZS11 and *green color* as an indicator of R11.

According to the method described above, a total of 14,658 recombination bin markers were obtained. Using the 14,658 recombination bin markers, we constructed a high-dense genetic map comprising of 19 chromosomes of AACC genome (10 AA and 9 CC) (**Table 3**, **Figure 2** and **Figure 3**). These total SNPs covered 2,198.85 cM with an average distance of 0.150 cM between the bin markers. The number of bin markers in each linkage group (LG) ranged from 231 (A10) to 1,517 (A03), with a mean of 771.47 bin markers per LG. The length of 19 LGs ranged from 87.31 cM (C01) to 170.72 cM (A05), with an average size of 115.73 cM. The average distance between adjacent bins ranged from 0.070 cM (A03) to 0.400 cM (C09). Most of the intervals between adjacent bins in the 19 LGs were less than 1 cM, and only 11 gaps with a distance of more than 1 cM were observed in eight LGs, including A03 (1), A06 (2), A09 (1), A10 (2), C03 (1), C05 (1), C07 (1), and C09 (2). The maximum gap was 7.143 cM on C09 (**Table 3**).

**Table 3:** Construction of a high-dense *B. napus* genetic map.

Linkage group	Bins	Map length (cM)	Average distance between loci (cM)	Max gap(cM)	Number of gaps >1 cM
A01	1,113	119.61	0.107	0.308	0
A02	294	108.93	0.371	0.715	0
A03	1,517	106.88	0.070	3.992	1
A04	1,157	116.05	0.100	0.279	0
A05	461	170.72	0.370	0.715	0
A06	883	100.09	0.113	4.942	2
A07	795	136.05	0.171	0.964	0
A08	544	120.83	0.222	0.617	0
A09	1,062	123.96	0.117	5.257	1
A10	231	89.65	0.388	1.072	2
C01	816	87.31	0.107	0.303	0
C02	825	128.59	0.156	0.300	0
C03	1,080	140.07	0.130	6.336	1
C04	727	100.54	0.138	0.391	0
C05	815	89.33	0.110	1.434	1
C06	635	126.13	0.199	0.383	0
C07	595	98.35	0.165	7.086	1
C08	827	123.27	0.149	0.428	0
C09	281	112.50	0.400	7.143	2
**Total/average**	**1,4658**	**2,198.85**	**0.150**	**7.143**	**11**

### QTL Mapping

**Figure 4:**
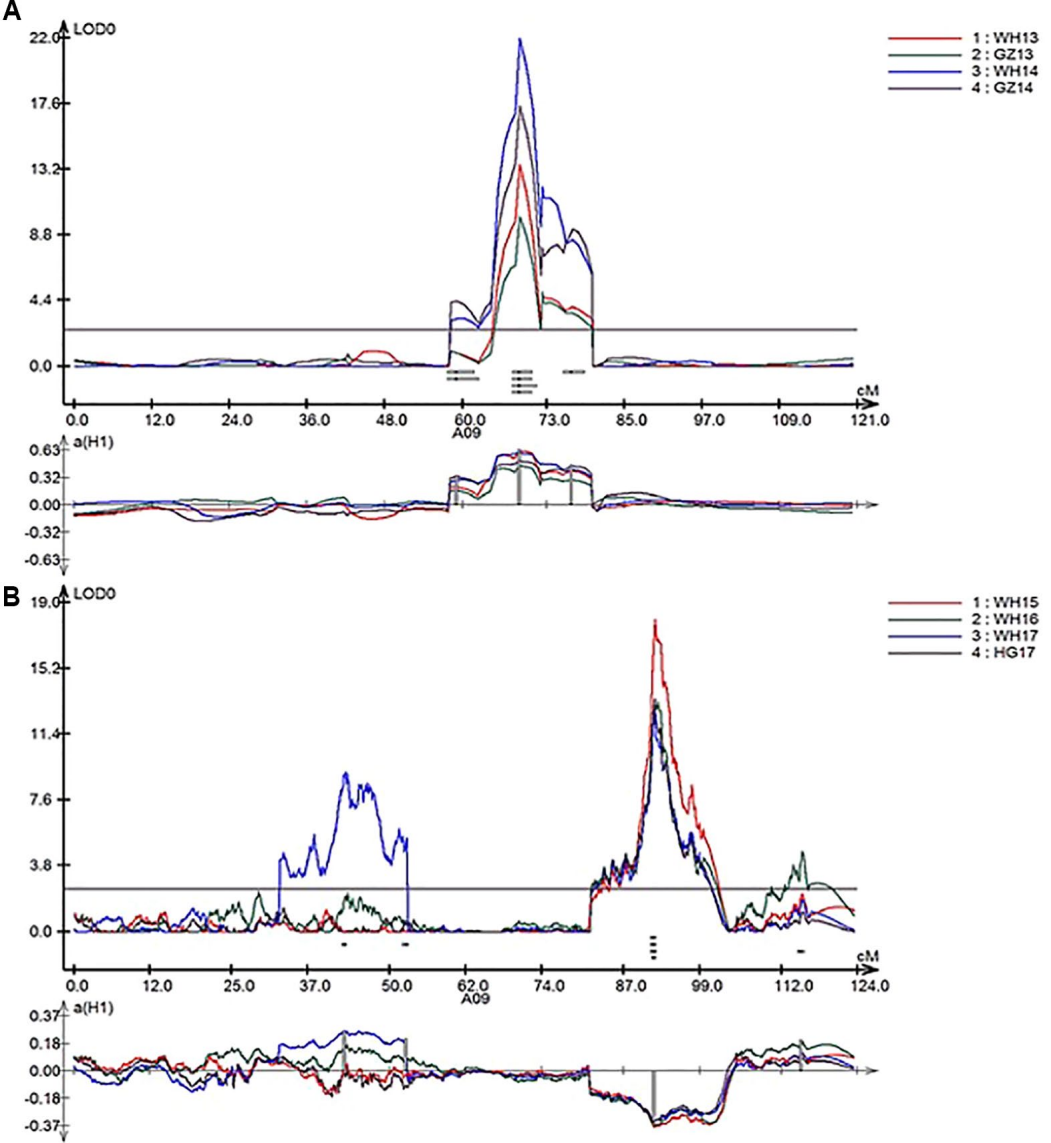
LOD profiles of RR **(A)** and ZR **(B)** populations for silique length (SL) as a major quantitative trait locus (QTL) in linkage group A09 from four environments.

In total, nine repeatedly detected QTL were identified for SL in RR and ZR populations (**Table 4**). Five QTL were frequently detected in different environments in RR population and were located on A01, A07, and A09. The explained phenotypic variance ranged from 3.28% to 39.07%, and the LOD values ranged from 2.53 to 21.99. A major QTL, *qSL_RR_A09b* (*R* ^2^ > 14%) was detected in all environments (**Table 4** and **Figure 4A**). Four QTL were repeatedly identified in different environments in ZR population and were located on A08, A09, C03, and C06. The explained phenotypic variance ranged from 3.01% to 20.36%, and the LOD values ranged from 2.57 to 17.50. A major QTL, *qSL_ZR_A09*, explaining more than 15% of the total SL variation was repeatly detected in all environments (**Table 4** and **Figure 4B**). Another QTL, *qSL_ZR_C06*, was also defined in all four environments and explained about 7% phenotypic variance in all environments. Different from RR population, two QTL for SL, including *qSL_ZR_A08* and *qSL_ZR_C03*, were repeatedly detected in two or three environments in ZR population.

**Table 4:** Repeatedly detected quantitative trait loci (QTL) for silique length (SL) in different environments in RR and ZR populations.

Population	QTL	Environment	Chromosome	Position	Closest marker	Confidence interval	LOD	Additive effect	*R* ^2^ (%)
RR	*qSL_RR_A01a*	WH13	A01	29.20	SNP02281A01	26.50–29.60	2.81	0.24	3.99
		ZY13	A01	29.20	SNP02281A01	28.00–29.40	5.85	0.33	8.63
	*qSL_RR_A01b*	ZY13	A01	40.10	SNP02208A01	39.30–40.60	3.54	0.26	6.37
		ZY14	A01	40.10	SNP02208A01	39.30–40.60	2.53	0.16	3.35
	*qSL_RR_A07*	ZY13	A07	91.50	SNP17353A07	90.80–92.30	2.67	0.18	3.28
		WH13	A07	91.50	SNP17353A07	85.10–92.50	2.85	0.21	4.02
	*qSL_RR_A09a*	WH14	A09	59.10	SNP20560A09	58.00–62.40	3.22	0.30	9.64
		ZY14	A09	59.10	SNP20560A09	58.00–61.70	4.36	0.33	12.58
	*qSL_RR_A09b*	ZY13	A09	68.80	SNP21007	68.10–70.10	10.05	0.46	14.99
		WH13	A09	68.80	SNP21007	68.10–69.80	13.55	0.63	25.69
		WH14	A09	68.80	SNP21007	68.10–69.80	21.99	0.61	39.07
		ZY14	A09	68.80	SNP21007	68.10–69.90	17.47	0.51	34.22
ZR	*qSL_ZR_A08*	WH15	A08	30.81	Block14756	30.40–31.00	14.33	0.31	15.89
		HG17	A08	30.81	Block14756	30.40–31.00	12.97	0.31	14.82
	*qSL_ZR_A09*	WH15	A09	91.74	Block16047	91.53–91.93	17.50	−0.37	20.36
		WH16	A09	91.74	Block16047	91.33–91.94	13.06	−0.35	15.18
		WH17	A09	91.74	Block16047	91.43–91.92	11.41	−0.34	15.00
		HG17	A09	91.74	Block16047	91.43–91.92	12.31	−0.34	15.42
	*qSL_ZR_C03*	WH16	C03	85.83	Block4097	84.92–86.02	3.00	0.15	3.02
		WH17	C03	85.53	Block4097	84.49–86.39	2.93	0.14	3.01
		HG17	C03	85.53	Block4097	84.09–86.59	2.57	0.13	2.65
	*qSL_ZR_C06*	WH15	C06	107.41	Block6910	107.00–108.40	6.77	0.21	7.22
		WH16	C06	107.81	Block6912	107.00–108.00	6.81	0.25	8.26
		WH17	C06	107.41	Block6910	107.00–107.60	9.52	0.28	10.99
		HG17	C06	107.21	Block6909	106.90–107.90	10.32	0.28	11.67

### Candidate Gene Identification

**Figure 5:**
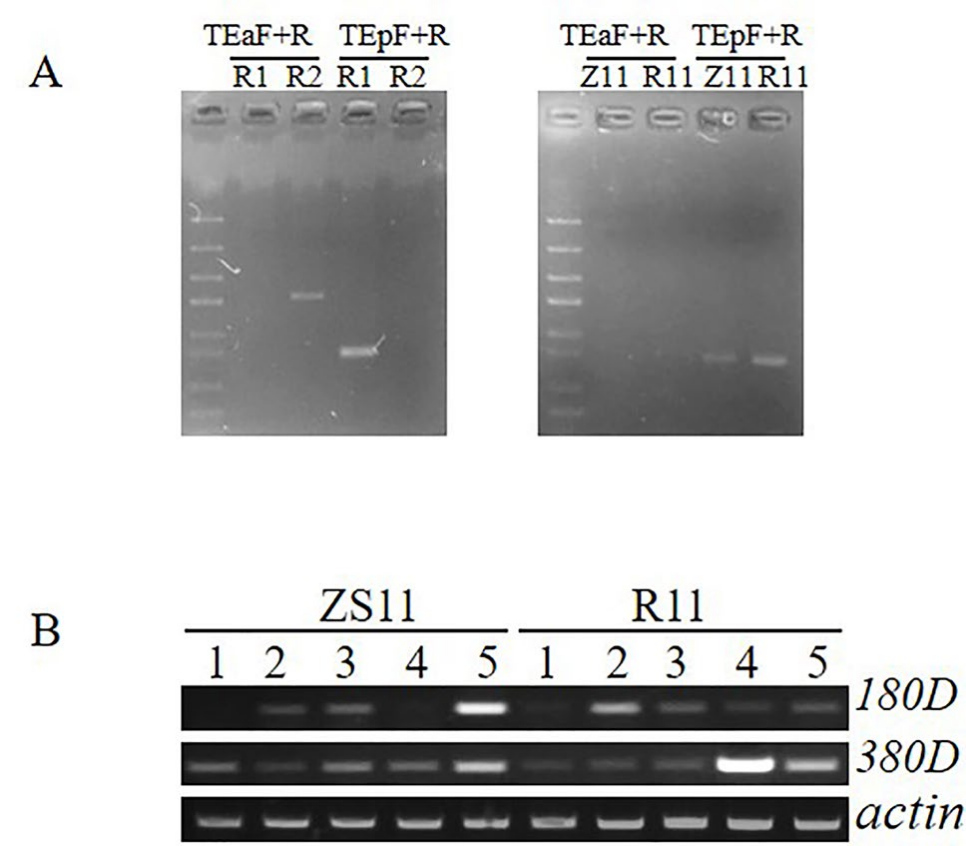
Detection of specific insertion of CACTA-like transposable element (TE) and expression comparison of two candidate genes. **(A)** The PAV PCR marker was used to detect the presence or absence of the 12.3-kb fragment and the presence or absence of the CACTA-like TE. TEa-F and TE-R specifically detect the presence of the 12.3-kb fragment and the absence of the TE, and the combination of TEp-F and TE-R specifically detects the presence of the TE and the absence of the 12.3-kb fragment. **(B)** Expression analysis between ZS11 and R11 in five tissue samples. *1* leaf, *2* stem, *3* flower bud with 2–2.5 mm, *4* flower bud with 3–3.5 mm, *5* young silique.

According to the results of QTL mapping, two major QTL, *qSL_RR_A09b* from RR population and *qSL_ZR_A09* from ZR population, were detected in all four environments. We then mapped the flanking sequences of the SNP markers in QTL confidence intervals to the *B. napus* reference genome (http://www.genoscope.cns.fr/brassicanapus/) using BLAST tool. The physical location of these two QTL was found on A09 between 27,577,590–28,622,867 bp and 28,619,958–28,994,184 bp, respectively (**Table 5**). This physical location overlaps with the SL QTL reported before (Dong et al., 2018). There are two genes, *BnaA9.ARF18* and *BnaA9.CYP78A9*, controlling SL cloned in *B. napus*; both are located on A09 (Liu et al., 2015; Shi et al., 2019). The CACTA-like TE which inserted into the upstream region of *BnaA9.CYP78A9* acting as an enhancer to increase SL (Liu et al., 2015; Shi et al., 2019) was detected in R1 (long silique), but not in R2 (short silique) (**Figure 5A**), suggesting *BnaA9.CYP78A9* might be the responsible gene for *qSL_RR_A09b* in RR population.

**Table 5:** Physical position analysis of the bin markers within the confidence interval of *qSL.A09*.

Population	Confidence interval (cM)	Marker	Genetic position (cM)	Physical position (bp)	Physical interval (bp)
RR	68.10–70.10	Bn-A09-p29756068	68.092	27,577,590	27,577,590–28,622,867
		Bn-A09-p30010889	68.379	27,836,100	
		Bn-A09-p30592138	68.668	28,375,060	
		Bn-A09-p30651428	68.769	28,434,345	
		Bn-A09-p30260475	69.218	28,037,360	
		Bn-A09-p30876251	69.887	28,621,746	
		Bn-A09-p30877372	70.240	28,622,867	
ZR	91.33–91.94	Block16042	91.261	28,619,958	28,619,958–28,994,184
		Block16043	91.366	28,623,615	
		Block16044	91.471	28,630,160	
		Block16045	91.576	28,641,282	
		Block16046	91.681	28,739,893	
		Block16047	91.786	28,834,253	
		Block16048	91.892	28,959,159	
		Block16049	91.997	28,994,184	

In contrast, *qSL_ZR_A09* in ZR population located in the physical interval of 28,619,958–28,994,184 was diverged from the QTL reported before, suggesting it is a novel QTL for SL control. To determine whether *BnaA9.ARF18* and *BnaA9.CYP78A9* were the SL genes for *qSL_ZR_A09* we further compared sequence variation between the two parental lines. No SNP was detected for *BnaA9.ARF18* sequence between two parents ZS11 and R11, and both ZS11 and R11 contained the 3.7-kb CACTA-like TE insertion (**Figure 5A**), excluding *BnaA9.ARF18* and *BnaA9.CYP78A9* as the responsible genes underlying *qSL_ZR_A09*. We then conducted candidate gene identification for this locus.

From the physical interval of *qSL_ZR_A09*, 78 candidate genes were found against the *B. napus* reference genome and 26 genes harbor SNPs or indels in gene or promoter region (**Supplemental File 1**, **Table S3**). According to the annotation from *Arabidopsis*, two candidate genes (*BnaA09g41180D* and *BnaA09g41380D*), respectively encoding homologs of *Arabidopsis* FLA3 and AGL61, which have been demonstrated to be involved in silique developmental regulation were identified. RT-PCR was performed to investigate the expression pattern of these two genes from five different tissue samples. The mRNA transcript of *BnaA09g41180D* was detected in flower buds, stem, and silique, but not in leaf sample. The *BnaA09g41380D* transcript was detected in all five tissue samples with various intensities (**Figure 5B**). The mRNA transcript of *BnaA09g41180D* was enriched more in ZS11 than R11 in young silique sample (**Figure 5B**). Fifty-one SNPs or indels were identified in the promoter region of *BnaA09g41180D* between ZS11 and R11. Among them, 42 including 31 SNPs and 11 Indels were located in the regulating *cis*-element of the promoter region. Thus, we speculated that *BnaA09g41180D* is the most promising candidate gene controlling SL at *qSL_ZR_A09*.

## Discussion

The *B. napus* genome was sequenced (Chalhoub et al., 2014) and various genetic maps had been published (Zhang et al., 2014), which enabled genotyping and provided a more effective method for exploring target regions with plenty of SNPs (Yang et al., 2012a; He et al., 2014). Traditional QTL prediction was mostly conducted by genotyping with a large number of individuals in a primary population, which is a time-consuming and painstaking work (Salvi and Tuberosa, 2005). In rapeseed, different techniques have been employed for QTL mapping (Uzunova et al., 1995; Zhao et al., 2006; Fu et al., 2007; Long et al., 2007; Radoev et al., 2008; Zhao et al., 2008; Shi et al., 2009; Yang et al., 2012b; Zhang et al., 2012; Raman et al., 2013; Wang et al., 2013; Raman et al., 2016; Yang et al., 2018). Identification of QTL by high-density SNP array analysis (Clarke et al., 2016) and re-sequencing (Fletcher et al., 2016) was proven a more efficient and quick method to ascertain the genomic regions of QTL. In this study, the ZR DH lines were re-sequenced and exhibited considerable variation in SL traits (**Figure 1**) and were ideal for genetic map construction (**Figure 2**) and QTL detection (**Figure 4**). The high-density genetic map developed in this study covered a total 2,198.85 cM with 14,658 recombination bin markers, indicating high-density markers for possible application in gene identification studies.

Four QTL for SL were identified located on A08, A09, C03, and C06 chromosomes in ZR DH population, explaining 3.01–20.36% phenotypic variation. Five QTLs located on A01 (2), A07, and A09 (2) were also detected in RR population, explaining 3.28 to 39.07% phenotypic variance. Major QTL was referred as QTL occurring at least once with *R* ^2^ ≥ 20% or at least twice with *R* ^2^ ≥ 10% (MacCaferri et al., 2008; Shi et al., 2009). Four QTL, *qSL_RR_A09b* occurring at four environments with *R* ^2^ from 14.99% to 39.07%, *qSL_ZR_A08* occurring at two environments with *R* ^2^ from 14.82% to 15.89%, *qSL_ZR_A09* occurring at four environments with *R* ^2^ from 15.00% to 20.36%, and *qSL_ZR_C06* occurring at two environments with *R* ^2^ from 10.99% to 11.67%, are all major QTL for SL.

Many QTL detected by this study indicated the polygenic nature of SL trait. Thus, different environment conditions such as rainfall, temperature, and soil may have some influence on the SL phenotype. As a matter of fact, our result on *qSL_RR_A09b* which explained 14.99–39.7% phenotypic variance also approved SL as a polygenic trait. This renders the difficulty in breeding for long siliques and the importance of marker assistant selection. Thus, the development of allele-specific markers for different loci is of great value for improving SL in breeding. For example, the favorable alleles of *qSL_ZR_A09* and *qSL_ZR_C06*, the two major QTL on A09 and C06 detected in ZR population, are from different parents. These favorable alleles can be pyramided using markers specific to the alleles and thus, longer SL can be readily acquired after pyramiding the different alleles from different loci.

We identified several QTL for SL including *qSL_ZR_A09* and *qSL_RR_A09b* in two genetic populations, revealing the possible existence of stable genetic loci on the A09 chromosome.

In previous SL QTL mapping studies, different QTL were identified on different LGs (Uzunova et al., 1995; Chen et al., 2007; Radoev et al., 2008; Xu et al., 2010; Yang et al., 2012b; Fu et al., 2015; Yang et al., 2017; Dong et al., 2018). One major effect QTL on A09 explained more than 50% variation as compared to a minor QTL revealing <10% variation (Yang et al., 2012b). Two genes, *BnaA.ARF18.a* and *BnaA9.CYP78A9*, have been demonstrated to be involved in the regulation of both SL and seed weight in rapeseed and are located on A09 (Liu et al. 2015; Shi et al., 2019). The QTL region of *qSL_RR_A09b* from RR population overlapped with *BnaA.ARF18.a* and *BnaA9.CYP78A9*. The CACTA-like transposable element inserted into the upstream region of *BnaA9.CYP78A9*, which contributes to the increase of SL, was found in R1 with long silique but not in R2 with short silique, indicating that QTL *qSL_RR_A09b* is identical to the QTL for seed weight (SW) and SL identified previously (Yang et al., 2012b; Li et al., 2014; Fu et al., 2015; Liu et al., 2015; Shi et al., 2019), and *BnaA9.CYP78A9* is the responsible gene for this locus.

The *qSL_ZR_A09* detected from ZR population showed a moderate effect (with *R* ^2^ value from 15% to 20.3%) in all environments, which could also be considered as major effect QTL. The physical region of *qSL_ZR_A09* was not overlapped with the QTL interval on A09 reported before. Both the parents (Z11 and R11) of ZR population contain the CACTA-like transposable element insertion (**Figure 5A**) and had no sequence variation in *BnaA.ARF18.a*. Thus, *qSL_ZR_A09* is very likely one novel QTL controlling SL. In the novel QTL region, two candidate genes (*BnaA09g41180D* and *BnaA09g41380D*) which are homologs of Arabidopsis FLA3 and AGL61, respectively. In *Arabidopsis*, modulation expression of FLA3 influenced elongation of the stamen filament and female fertility, which led to the change of silique length (Li et al., 2010). Siliques of the heterozygous mutant of *agl61* are smaller than the wild-type siliques and seed set was also reduced (Bemer et al., 2008). The mRNA transcript of *BnaA09g41180D* was increased in ZS11 compared to R11 in young silique sample. Many SNPs or indels were identified in the promoter region of *BnaA09g41180D* between ZS11 and R11, with most of the sequence variation in the promoter region. The variation regulating *cis*-element in the promoter region may lead to differential expression level of candidate genes. Thus, we considered that *BnaA09g41180D* is the most promising candidate gene for *qSL_ZR_A09*.

In summary, we have developed two DH populations and conducted high-density SNP maps to identify novel and major QTL for SL, which is one of the most important yield components. Two major QTL on A09 were identified and candidate genes were predicted. One of the major QTL identified is probably a previously identified SL gene and the other one is a newly found SL gene in *B. napus*. Sequence variation of these genes ensures the development of gene-specific molecular markers and thus will facilitate pyramiding of favorable alleles of these genes *via* MAS in the breeding program for yield increase. As both identified QTL are located on A09, this result may be helpful for breeding of other *Brassica* species including *B*. rapa and *Brassica* juncea. Specific markers developed for the main effect QTL in this study will be useful for marker-assisted selection in breeding. Main effect QTL detected in the population will also provide target for map-based cloning of genes controlling silique length. Sequence variation of candidate genes will ensure the development of gene-specific markers and thus facilitate pyramiding breeding.

## Data Availability Statement

The datasets generated for this study can be found in NCBI https://www.ncbi.nlm.nih.gov/bioproject/PRJNA588593/. 

## Author Contributions

HW and QH designed the experiment. HW, WH, LF, and DM contributed to phenotypic measurements. WW, HC, and JL contributed to data analysis. BD and MH contributed to the expression and gene analysis. HW and QZ wrote the manuscript. HC and QH revised the manuscript. All authors reviewed and approved this submission.

## Conflict of Interest

The authors declare that the research was conducted in the absence of any commercial or financial relationships that could be construed as a potential conflict of interest.
