# Supplementary material for: QTL and Candidate Gene Identification for Silique Length Based on High-Dense Genetic Map in Brassica napus L
Source: Front Plant Sci. 2019 Nov 29;10:1579. doi: 10.3389/fpls.2019.01579 (PMC6895753; doi:10.3389/fpls.2019.01579)
Supplement: Supplementary file 2 [file Presentation_1.pdf]

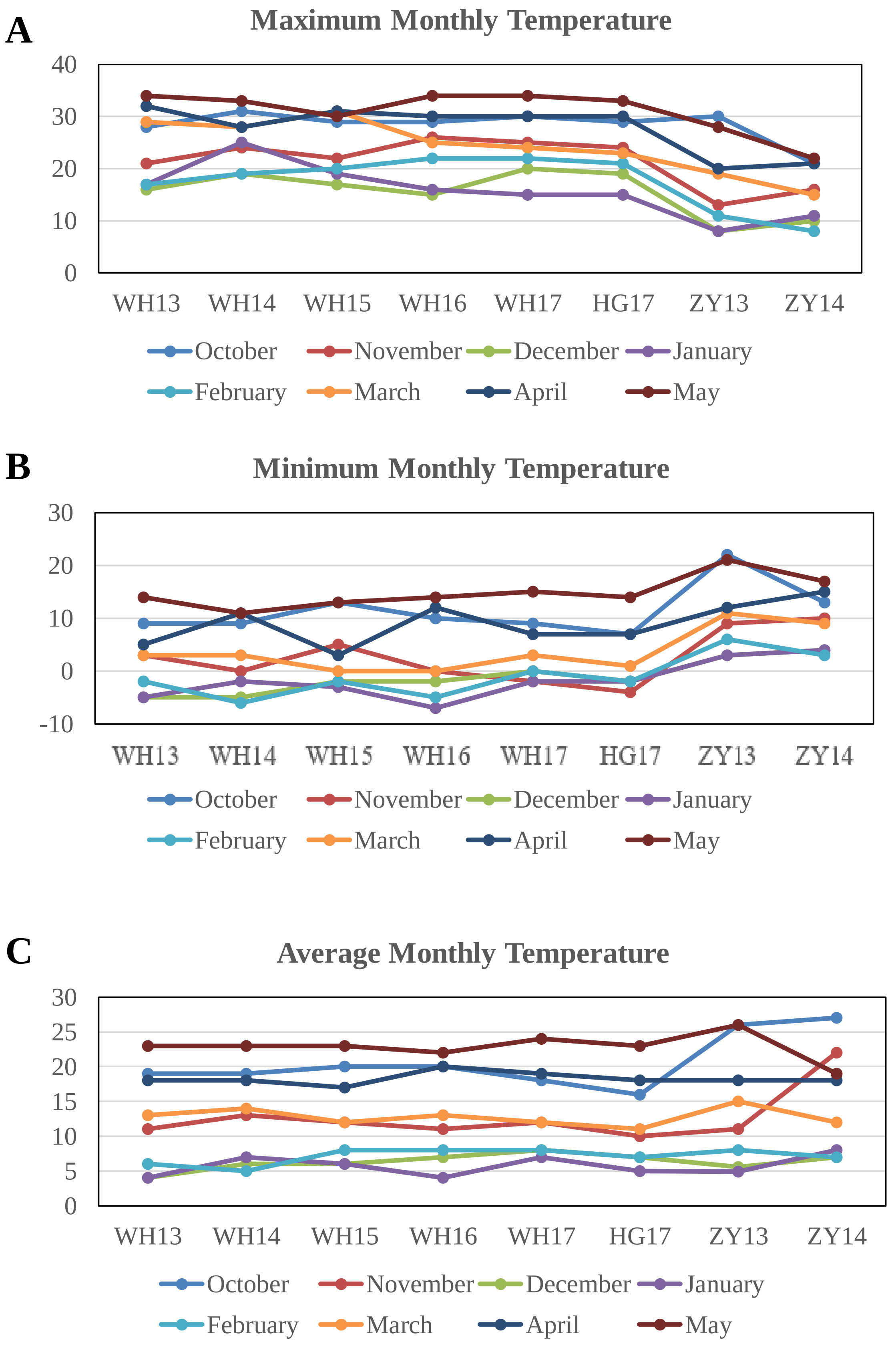


**Figure S1: Monthly temperature of growing season in Wuhan (2013-17), Huanggang (2017)and Zunyi (2013-14). A),** Maximum monthly temperature of different growing season. **B),** Depicts minimum monthly temperature of all growing season **C),** Exhibit average monthly temperature of all growing season.
